# Editorial to predictors of the need for atrioventricular nodal ablation following redo ablation for atrial fibrillation

**DOI:** 10.1002/joa3.13041

**Published:** 2024-04-17

**Authors:** Takatsugu Kajiyama, Yusuke Kondo, Yoshio Kobayashi

**Affiliations:** ^1^ Department of Advanced Arrhythmia Bioengineering Chiba University Graduate School of Medicine Chiba Japan; ^2^ Department of Cardiovascular Medicine Chiba University Graduate School of Medicine Chiba Japan

Editorial to predictors of the need for atrioventricular nodal ablation following redo ablation for atrial fibrillation.^1^

In patients with atrial fibrillation (AF) or atrial tachycardia (AT), achieving an optimal rate control is essential for improving the outcomes and enhancing the quality of life. Beta‐blockers or calcium channel blockers are commonly used to significantly reduce the ventricular response. However, a subset of AF patients may experience an inadequate rate control even after receiving the maximum‐tolerated dose of bradycardic agents. Catheter ablation is one of the effective options, but its success rate varies among patients. For example, in patients with hypertrophic cardiomyopathy, additional catheter ablation after the first ablation exhibits a low success rate below 50%.^2^, ^3^ Furthermore, recurrent ATs can often trigger a rapid ventricular response more easily than AF, presenting significant challenges for the diagnosis and treatment due to factors such as epicardial bridges or complex circuits during catheter ablation. In such cases, atrioventricular nodal ablation (AVNA) combined with the simultaneous implantation of a pacing device has been established as a viable solution.^4^ AVNA is reportedly effective in improving symptoms,^5^ functional capacity,^6^ and echocardiographic parameters.^7^, ^8^ The main advantage of the AVNA is that its therapeutic effect is less uncertain than medications and catheter ablation. The heart rate is completely regulated by the pacemaker after the AVNA, and reconduction of the intrinsic conduction is rare. Moreover, the recent advancements in physiological pacing techniques, such as para‐Hisian pacing, left bundle branch area pacing, and biventricular pacing, have made AVNA more appealing by reducing the risk of pacing‐induced cardiomyopathy. The ability to control and regularize the heart rate after the AVNA is advantageous for maximizing the cardiac output and minimizing the patient symptoms. The symptomatic, echocardiographic, and functional benefits of AVNA have been reported in multiple reports. If some AF is refractory to repeated catheter ablation procedures, AVNA might offer a substantial benefit not only from the patient's perspective but also from an economic standpoint.

In the original investigation in this issue of the Journal of Arrhythmia, Calvert et al. identified a female sex, ischemic heart disease, preexisting pacemakers, and persistent AF as predictors of an AVNA after a second attempt at catheter ablation of AF. As mentioned above, catheter ablation of AF does not always meet the clinical expectations, leaving room for considering an AVNA as an alternative and more reliable treatment, albeit more invasive. If the physicians acknowledge the clinical predictors of an AVNA before a second session with limited efficacy, it should contribute to reducing any unnecessary treatment. From this point of view, the present investigation may be valuable in clinical practice.

The readers should note the limitations acknowledged by the authors. The conclusions are based on retrospective data from a single institution. Therefore, the therapeutic decisions and patient stratification observed may not be directly applicable to other centers.

While the recent advancements in catheter ablation of AF are indeed remarkable, it remains crucial to carefully balance the potential benefits and reliability of each therapeutic option. There is a need for further research to accurately identify the patients who would most benefit from an AVNA. Such investigations will help refine the patient selection criteria, ensuring those who undergo an AVNA receive the maximum clinical benefit.

## CONFLICT OF INTEREST STATEMENT

Authors declare no conflict of interests for this article.
